# Treg-Therapy Allows Mixed Chimerism and Transplantation Tolerance Without Cytoreductive Conditioning

**DOI:** 10.1111/j.1600-6143.2010.03018.x

**Published:** 2010-04

**Authors:** N Pilat, U Baranyi, C Klaus, E Jaeckel, N Mpofu, F Wrba, D Golshayan, F Muehlbacher, T Wekerle

**Affiliations:** a Division of Transplantation, Department of Surgery, Medical University of ViennaAustria; b Department of Gastroenterology, Hepatology and Endocrinology, Hannover Medical School (MHH)Hannover, Germany; c Institute of Clinical Pathology, Medical University of ViennaAustria; d Transplantation Centre and Transplantation Immunopathology Laboratory, Centre Hospitalier Universitaire Vaudois (CHUV), Lausanne UniversityLausanne, Switzerland

**Keywords:** Mixed chimerism, tolerance, transplantation

## Abstract

Establishment of mixed chimerism through transplantation of allogeneic donor bone marrow (BM) into sufficiently conditioned recipients is an effective experimental approach for the induction of transplantation tolerance. Clinical translation, however, is impeded by the lack of feasible protocols devoid of cytoreductive conditioning (i.e. irradiation and cytotoxic drugs/mAbs). The therapeutic application of regulatory T cells (Tregs) prolongs allograft survival in experimental models, but appears insufficient to induce robust tolerance on its own. We thus investigated whether mixed chimerism and tolerance could be realized without the need for cytoreductive treatment by combining Treg therapy with BM transplantation (BMT). Polyclonal recipient Tregs were cotransplanted with a moderate dose of fully mismatched allogeneic donor BM into recipients conditioned solely with short-course costimulation blockade and rapamycin. This combination treatment led to long-term multilineage chimerism and donor-specific skin graft tolerance. Chimeras also developed humoral and *in vitro* tolerance. Both deletional and nondeletional mechanisms contributed to maintenance of tolerance. All tested populations of polyclonal Tregs (FoxP3-transduced Tregs, natural Tregs and TGF-β induced Tregs) were effective in this setting. Thus, Treg therapy achieves mixed chimerism and tolerance without cytoreductive recipient treatment, thereby eliminating a major toxic element impeding clinical translation of this approach.

## Introduction

Outcome after organ transplantation could be improved through the establishment of donor-specific immunological tolerance. Rodent models of tolerance through mixed chimerism are among the most robust and are thus attractive candidates for clinical development (1–8). The exceptional clinical potential of the mixed chimerism approach has recently been underscored by a pilot series of renal transplant recipients cotransplanted with donor BM who accepted their organ graft in most cases without maintenance immunosuppression (9). Routine clinical application of this approach, however, is prevented by the toxicity of the cytoreductive recipient conditioning which is required to allow even transient engraftment of MHC-mismatched BM.

Since the concept's introduction with myeloablative conditioning (10), gradually less toxic murine mixed chimerism regimens have been developed. However, the induction of durable mixed chimerism in robust strain combinations has universally required either recipient irradiation (2,3,6,11), cytotoxic drug/antibody treatment (8,12,13) or clinically unobtainable high doses of BM (4,14,15). As irradiation and cytotoxic drugs are associated with considerable medical risks such as profound leukopenia (9), their elimination from tolerance protocols is widely regarded as essential for widespread clinical translation. This goal of a noncytoreductive mixed chimerism model with conventional BM doses has remained elusive so far.

Tregs play a critical role in maintaining self-tolerance. The therapeutic exploitation of Tregs has pronounced effects in autoimmune (16), organ transplantation (17,18) and GVHD models (19,20), but does not induce skin graft tolerance across full MHC barriers on its own (11,17), which is regarded as a stringent test in rodent models. Numerous naturally occurring and experimentally designed subsets of Tregs have been described that are being considered for clinical application (21). Through transduction with FoxP3, large numbers of Tregs (denoted here FoxP3-Tregs), whose properties resemble those of natural Tregs, can be generated for experimental purposes (22,23). Although the role of FoxP3 in humans is more complex (24), lentiviral transduction of FoxP3 has recently allowed generation of stable human Tregs (23). Induction of Tregs through *in vitro* exposure of murine T cells to TGF-β (iTregs) likewise allows the production of large quantities of Tregs (25,26). Natural CD4^+^CD25^+^ Tregs (nTregs) are currently already under evaluation in several clinical trials (27).

We therefore investigated the therapeutic potential of several populations of Tregs (FoxP3-Tregs, nTregs and iTregs) to induce engraftment of conventional doses of allogeneic BM, mixed chimerism and transplantation tolerance without cytoreductive recipient conditioning.

## Materials and Methods

### Animals

Female C57BL/6 (B6, recipient, H-2^b^), Balb/c (donor, H-2^d^) and C3H/N (third party, H-2^k^) mice were purchased from Charles River Laboratories (Sulzfeld, Germany). This donor-recipient strain combination is one of the most stringent models as it crosses MHC mismatches plus minor histocompatibility antigen mismatches, and as B6 recipients are relatively costimulation blockade-resistant (13,28). All mice were housed under specific pathogen-free conditions and were used at 6 to 12 weeks of age. All experiments were approved by the local review board of the Medical University of Vienna, and were performed in accordance with national and international guidelines of laboratory animal care.

### Generation of tregs

For *FoxP3-Tregs*, vector pCMMP-FoxP3-IRESeGFP (FoxP3/GFP) or control vector pCMMP-IRESeGFP (GFP), vector pMD containing viral proteins gag and pol and packaging vector pMD.G encoding for VSV-G protein (16) or K73 encoding for an ecotrophic envelope (kindly provided by Dr. Christopher Baum, Hannover Medical School, Germany) were transfected into 293 T cells, retroviral supernatant was recovered, concentrated by ultracentrifugation and viral titer was determined. For retroviral infection CD4^+^ cells were isolated from B6 spleen and lymph nodes by magnetic bead sorting (L3T4 microbeads; Miltenyi Biotec, Bergisch Gladbach, Germany), and were cultured in plates coated with 10 μg/mL anti-CD3 (145–2C11), 1 μg/mL anti-CD28 (37.51) (BD Pharmingen, San Jose, CA) and in the presence of 100 U/mL rmIL-2 (Sigma-Aldrich, St. Louis, MO). Cells were infected twice (d2 and 3) with high-titer VSV-G pseudotyped retrovirus at a multiplicity of infection of 5–10 as described previously (16). Cells were FACS sorted for GFP expression on d4. We achieved transduction efficiencies of up to 48%. The vector contained FoxP3 followed by an internal ribosomal entry site for bicistronic GFP expression. In some experiments, an ecotrophic envelope instead of VSV-G protein was used, resulting in higher transduction efficiencies.

*nTregs* were isolated from spleen and lymph nodes of naïve B6 mice. CD4^+^CD25^+^ cells were purified by magnetic bead separation using negative selection for CD4^+^ and subsequent positive selection of CD25^+^ by incubation with PE-conjugated anti-CD25 (7D4) followed by anti-PE microbeads (CD4^+^CD25^+^ Regulatory T-cell Isolation Kit; Miltenyi Biotec). Purity of separated cells was >90%. Cells were used *in vivo* after cultivation for 5 days in plates coated with 10 μg/mL anti-CD3 (145–2C11) and 1 μg/mL anti-CD28 (37.51) (BD Pharmingen) in the presence of 100 U/mL IL-2 (Sigma).

For generation of *iTregs*, CD4^+^ cells were isolated from B6 spleen and lymph nodes by magnetic bead sorting (L3T4 microbeads; Miltenyi Biotec), and were cultured for 5 days in plates coated with 10 μg/mL anti-CD3 (145–2C11), 1 μg/mL anti-CD28 (37.51) (BD Pharmingen) in the presence of 100 U/mL IL-2 (Sigma) and 5 ng/mL rhTGFbeta (R&D Systems, Minneapolis, MN) (26). All Treg populations were phenotypically characterized by flow cytometry before *in vivo* use.

### Suppression assay and mixed lymphocyte reaction (MLR)

The 4 × 10^5^ B6 responder cells (unseparated splenocytes) were cocultured with escalating numbers of FoxP3-transduced Tregs (2 × 10^5^, 4 × 10^5^, 8 × 10^5^, for a ratio of 2:1, 1:1, 1:2, respectively) or freshly sorted CD4^+^CD25^high^ Tregs respectively, in the presence of 4 × 10^5^ irradiated (30 Gy) Balb/c stimulator cells (unseparated splenocytes). Cells were pulsed with [3H]-thymidine (Amersham, Biosciences, UK) for 18 h after 72 h of incubation. Incorporated radioactivity was measured using scintillation fluid in a ß-counter. Stimulation indices (SI) were calculated in relation to medium controls. MLRs were performed with unseparated splenocytes as described previously (4).

### BMT protocol

Groups of age-matched B6 recipients received costimulation blockade consisting of anti-CD40L (CD154) mAb (MR1, 1 mg, d0) and CTLA4Ig (0.5 mg, d2) (3), a short course of rapamycin (0.1 mg/mouse, d-1, d0 and d2) (Alexis Biochemicals, San Diego, CA) (6) and approximately 2 × 10^7^ unseparated BM cells recovered from Balb/c donors (d0, i.v.) with or without additional Treg treatment. Treg treatment consisted of 4 × 10^6^*FoxP3-Tregs* (d0) or 3 × 10^6^*nTregs* (d0) or 5 × 10^6^*iTregs* (d0). Anti-CD154 mAb was purchased from BioXCell (West Lebanon, NH), hCTLA4Ig (abatacept) was generously provided by Bristol-Myers, Squibb Pharmaceuticals (Princeton, NJ).

### Secondary BMT

Eight weeks after BMT, BM cells were recovered from primary recipients and transplanted into secondary B6 mice conditioned with 10 Gy total body irradiation (TBI), depleting doses of anti-CD8 (2.43; 0.5 mg/mouse) and anti-CD4 (GK1.5; 0.5 mg/mouse) mAbs and anti-CD40L mAb (MR1; 0.5 mg/mouse) to promote engraftment. On the day of reconstitution each secondary recipient was transplanted with 5 × 10^7^ BM cells recovered from one chimera (i.v.).

### Antidonor antibodies

Recipient serum recovered 1 week, 2 weeks and >3 months post-BMT was heat-inactivated and incubated with recipient- and donor-type thymocytes (which are low in Fc-receptors, reducing background). Binding of serum IgG Abs to thymocytes was analyzed by flow cytometry using FITC-conjugated rat anti-mouse IgG1 and IgG2a/2b (BD Pharmingen).

### Flow cytometric analysis of Treg phenotype, chimerism and deletion

Multicolor flow cytometric analysis of Treg phenotype, multilineage chimerism and Vβ-subunit expression was performed as described previously (3). Chimerism was calculated as the net percentage of donor MHC class I^+^ (H-2D^d^, 34-2-12) cells among leukocyte lineages, as described previously (3,6). Mice were considered chimeric if donor cells were detectable by flow cytometry within both the myeloid lineage and at least one lymphoid lineage. For analysis and sorting of Tregs, mAbs with specificity against CD4 (RM4-4), CD25 (7D4) and CD62L (l-selectin, Mel-14) were used. For intracellular staining, a FoxP3 (FJK-16s) staining Kit (eBioscience, San Diego, CA) was used according to the manufacture's protocol. Cell sorting was performed on a FACS Aria (BD Biosciences, San Jose, CA), purity of sorted populations was >95%.

### Histological analysis

Four micrometers sections were cut from paraffin-embedded tissue fixed in 4.5% formalin (with a buffered pH of 7.5), stained with hematoxilin-eosin and Giemsa according to standard protocols, and analyzed by an experienced pathologist. Mast cells were counted in five high-power fields (HPF, magnification 400×), and mean density per HPF was calculated. For immuno-histochemistry, 6 μm sections were prepared and biotinylated anti-Foxp3 (clone FJK-16S, eBioscience) followed by Streptavidin/HRP (Dako, Glostrup, Denmark) was used.

### Isolation of genomic DNA and PCR

DNA was isolated from skin grafts, lymph nodes, BM, spleen, thymus and FoxP3-transduced cells using the DNeasy Blood & Tissue kit (Qiagen, Hilden, Germany). GFP product was amplified using primer 5′-CGCACCATCTTCTTCAAGGACGAC-3′ and primer 5′-AACTCCAGCAGGACCATGTGATCG-3′. FoxP3 transduced NIH3T3 cells and retroviral vector were used as positive controls, β-actin DNA was amplified as an internal control (16).

## Results

### Phenotypic and functional characteristics of FoxP3-Tregs, nTregs and iTregs

**Figure 1:**
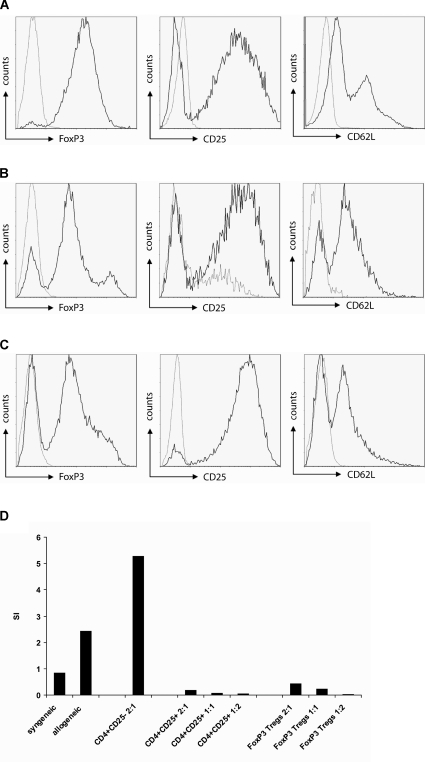
Phenotypical and functional characterization of FoxP3-Tregs, nTregs and iTregs. B6 CD4^+^ cells were transduced with FoxP3/GFP virus and sorted for GFP expression to generate FoxP3-Tregs (A) or *in vitro* cultured with TGFβ to yield iTregs (C). B6 CD4^+^CD25^+^ nTregs (B) were isolated from spleen and lymph nodes and activated *in vitro*. Prior to *in vivo* transfer, cells were phenotypically characterized by flow cytometry. All three populations expressed high levels of FoxP3, CD25 and CD62L. Typical histograms (gated on CD4^+^ cells) for intracellular expression of FoxP3 and surface expression of CD25 and CD62L are shown. (D) FoxP3-Tregs suppressed proliferation of B6 splenocytes in response to allogeneic stimulation (Balb/c splenocytes) (as do freshly sorted control CD4^+^CD25^+^ Tregs), whereas coculture with CD4^+^CD25^−^ sorted cells resulted in enhanced proliferation. SI (stimulation index) was calculated by dividing the mean cpm from responses against syngeneic (B6) or allogeneic (Balb/c) stimulator cells by mean background cpm.

Three different populations of polyclonal recipient-type Tregs were generated for *in vivo* use. Polyclonal *FoxP3-Tregs* were produced by retroviral transduction of wild-type B6 CD4^+^ lymphocytes with a retroviral vector containing FoxP3 followed by an internal ribosomal entry site (IRES) for direct GFP translation (16). CD4^+^CD25^+^*nTregs* were separated from B6 spleen and lymph nodes and transferred *in vivo* upon polyclonal *in vitro* activation. For generation of *iTregs*, B6 CD4^+^ cells were cultured *in vitro* in the presence of TGF-β and IL-2 (26). All three cell populations showed a phenotypic pattern characteristic of Tregs, including expression of high levels of FoxP3, CD25 and CD62L (Figure 1A–C). Little is known about the ability of *polyclonal* FoxP3-Tregs to suppress alloreactivity across MHC barriers, in particular as they were ineffective in a minor-mismatch-only heart transplant model (29). We thus first assessed their regulatory function *in vitro*. Coculture assays revealed that FoxP3-Tregs suppressed proliferation of T cells in response to alloantigen in a dose-dependent manner (Figure 1D) (similar results were obtained for iTregs; data not shown).

### Treg treatment leads to mixed chimerism without cytoreductive recipient conditioning

**Figure 2:**
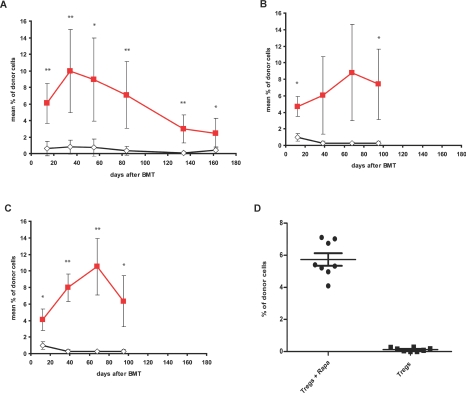
Treg treatment together with rapamycin induces mixed chimerism without cytoreductive conditioning. Groups of B6 mice were grafted with 2 × 10^7^ Balb/c BM cells under the cover of costimulation blockade (anti-CD154, CTLA4Ig) and rapamycin and were additionally treated (A) with (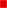, 4 × 10^6^, n = 7) or without (◊, n = 6) recipient-derived FoxP3-Tregs, (B) with (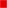, 3 × 10^6^, n = 4) or without (◊, n = 7) recipient-derived nTregs or (C) with (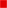, 5 × 10^6^, n = 6) or without (◊, n = 7) recipient-derived iTregs, respectively. Only recipients treated with any of the Treg populations developed chimerism. Donor (H-2D^d+^) chimerism among leukocytes of the myeloid (Mac1^+^) lineage was assessed by flow cytometry of peripheral blood at multiple time points and is shown as mean percent (error bars indicate standard deviation; nTreg and iTreg groups were done in the same experiment, therefore the control group is shown twice in B + C). Repeat experiments performed with nTregs and iTregs showed similar results. **p < 0.005, *p < 0.05 with versus without Tregs (two-sided Student's t-test). (D) B6 mice received 2 × 10^7^ Balb/c BM cells under the cover of costimulation blockade and 5 × 10^6^ iTregs with or without rapamycin. Recipients treated without rapamycin failed to develop chimerism (• iTregs with rapamycin n = 8; 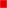 iTregs without rapamycin, n = 8; p = 0.0002, Fisher's exact test). Donor (H-2D^d+^) chimerism among leukocytes of the myeloid (Mac1^+^) lineage is shown 2 weeks post-BMT as scatter plot (mean and standard deviation are indicated).

Next, the three Treg populations were used *in vivo* as part of a BMT regimen. Without any irradiation or cytotoxic drug/antibody conditioning, B6 mice received a conventional dose of fully mismatched Balb/c BM (2 × 10^7^ cells per mouse) under the cover of costimulation blockade (CTLA4Ig, anti-CD40L mAb) and a brief course of rapamycin. Rapamycin was used as it has an engraftment-enhancing effect (which, however, is insufficient for engraftment of such a BM dose without recipient irradiation, as shown by the results of the control groups depicted in Figure 2A–C) (6) and positively affects Treg function *in vitro* and *in vivo* (30–32). Treg treatment (3 × 10^6^ to 5 × 10^6^ cells/recipient) led to long-term mixed chimerism in the vast majority of BMT recipients whereas no BMT recipient developed chimerism without Treg treatment (6/7 long-term chimeras [plus one transient chimera] with FoxP3-Tregs; 3/4 chimeras with nTregs, 5/6 chimeras with iTregs; 0/13 chimeras without Tregs; at 3–7 months post-BMT; p = 0.0002 for FoxP3-Tregs; p = 0.0059 for nTregs; p = 0.0005 for iTregs; Fisher's exact test; independent repeat experiments performed for iTreg and nTreg treatment showed similar results). BMT recipients treated with Tregs reached substantial levels of macro-chimerism (mean blood chimerism levels ∼3 months post-BMT among the myeloid lineage were 5–10%) (Figure 2A–C). Rapamycin was indispensable as adjunctive treatment given together with Tregs, as mice receiving Tregs without rapamycin uniformly failed to develop chimerism (8/8 chimeras with rapamycin; 0/8 without rapamycin; p = 0.0002, Fisher's exact test; 2 weeks post-BMT) (Figure 2D). Thus, the combination of Tregs with rapamycin—but not either alone—allowed BM engraftment without cytoreduction.

**Figure 3:**
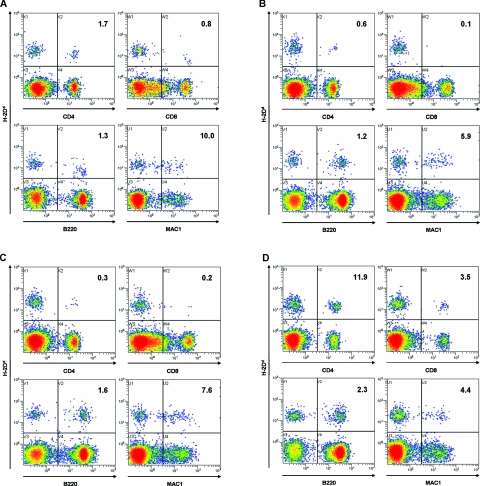
Treg treatment leads to multilineage chimerism and stem cell engraftment. Chimerism in Treg-treated mice was of multilineage nature as shown by the presence of donor cells among the T-cell (CD4, CD8), B-cell (B220) and myeloid (Mac1) lineages. Two-color flow cytometry plots are shown from representative BMT recipients ∼3 months post-BMT treated with (A) FoxP3-Tregs, (B) nTregs and (C) iTregs. (D) BM was recovered from iTreg-treated chimeras (8 weeks post-BMT; n = 3) and transplanted into myoablated secondary B6 recipients. Donor chimerism was detectable in the T-cell (CD4, CD8), B-cell (B220) and myeloid (Mac1) lineages 12 weeks postsecondary BMT, indicating that donor hematopoietic stem cells had engrafted in primary recipients. Flow cytometry plots are shown from one secondary recipient. Numbers indicate the net percentage of donor chimerism in the depicted lineage (for calculation algorithm, please see section ‘Materials and Methods’).

Chimerism in Treg-treated BMT recipients was of multilineage nature, with donor populations present in all tested leukocyte lineages, including CD4 and CD8 cells (Figure 3A–C). Chimerism levels in peripheral blood correlated with chimerism in lymphoid organs (BM and spleen, data not shown). Multilineage chimerism persisted for the length of follow up (>5 months post-BMT), suggesting that donor hematopoietic stem cells had successfully engrafted and survived in recipient mice (33). To assess more directly whether hematopoietic stem cells had successfully engrafted, BM recovered from iTreg-treated chimeras 8 weeks post-BMT was transplanted into myeloablated secondary B6 recipients (n = 3). Long-term multilineage donor chimerism was detectable in 2/3 secondary recipients (15.3% mean CD4 chimerism, 5.8% mean CD8 chimerism, 2.4% mean B-cell chimerism, 6.0% mean myeloid chimerism; 12 weeks postsecondary BMT), suggesting that donor hematopoietic stem cells had indeed successfully engrafted and survived in the primary recipients (Figure 3D). Thus, allogeneic stem cell engraftment and long-lasting multilineage chimerism was induced through Treg therapy without cytoreductive recipient conditioning.

### Chimeras induced through Treg treatment demonstrate skin graft tolerance

**Figure 4:**
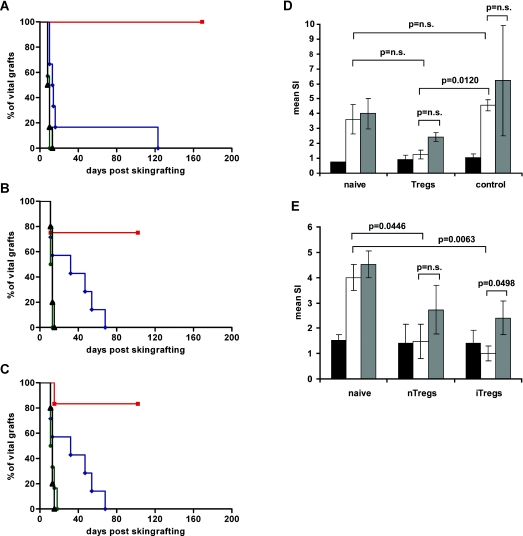
Chimeras induced through Treg treatment develop donor-specific skin graft tolerance and hyporesponsiveness *in vitro*. Donor-specific tolerance was assessed by grafting of full thickness donor and third party (C3H) tail skin 4–6 weeks post-BMT. Grafts were considered to be rejected when less than 10% remained viable. Donor skin graft survival was significantly prolonged in BMT recipients treated with (A) FoxP3-Tregs (▪, n = 7; p = 0.0002) compared to BMT recipients without Tregs (♦, n = 6); and in recipients treated with (B) nTregs (▪, n = 4; p = 0.0360) and (C) iTregs (▪, n = 6; p = 0.0043) compared to the controls without Tregs (♦, n = 7, same control group for B + C). Third-party grafts were promptly rejected in all groups (Treg-treated •, control ▵). Survival was calculated according to the Kaplan–Meier product limit method and compared between groups using the log-rank test. Repeat experiments performed with nTregs and iTregs showed similar results. (D,E) Mixed lymphocyte reaction results from selected BMT recipients were obtained early (1 week post-BMT) and at the end of follow-up (22 weeks post-BMT). (D) Early MLRs (1 week post-BMT) demonstrate hyporesponsiveness toward the donor in Treg-treated recipients (n = 2) in comparison to controls without Tregs (p = 0.0120, SI antidonor compared to controls). (E) Long-term chimeras of Treg-treated mice (nTreg, n = 2; iTreg, n = 3) showed specific hyporesponsiveness toward the donor *in vitro* (p = 0.0446 for nTregs; p = 0.0063 for iTregs, SI antidonor compared to naïve B6 mice). SIs were calculated by dividing the mean cpm from responses against recipient (black column; B6), donor (white column; Balb/c), or third-party (gray column; C3H) stimulator cells by mean background cpm (i.e. cpm with no stimulator population). Error bars indicate standard deviation.

Donor skin graft survival is commonly regarded as a stringent test for assessing transplantation tolerance. We therefore grafted BMT recipients with donor and third party skin (4–6 weeks post-BMT). Most mice treated with Tregs, but no mouse without Tregs, accepted donor skin for the length of follow-up, and all mice promptly rejected third party grafts (7/7 donor skin graft acceptors with FoxP3-Tregs; 3/4 with nTregs, 5/6 with iTregs vs. 0/13 without Tregs, p < 0.01 for each Treg group, Fisher's exact test; MST [median survival time] >100 days for donor grafts of each Treg group; MST = 14 without Tregs; third-party C3H skin grafts were rejected within 15 days in all groups) (Figure 4A–C). The same regimen without BM (iTregs, costimulation blockade and rapamycin) failed to induce skin graft tolerance (n = 6; MST = 20 days; not shown). Hence, combining Treg treatment with BMT leads to donor-specific skin graft tolerance.

### Chimeras induced through Treg treatment show humoral and *in vitro* tolerance

Development of antidonor Abs (antibodies) is associated with the occurrence of chronic rejection and late graft loss in clinical transplantation and suggests incomplete tolerance in the experimental setting (34). Hence we tested BMT recipients for the presence of antidonor antibodies early (1 and 2 weeks) and late (>3 months) after BMT through flow cytometric assessment of recipient serum binding to donor cells. No antidonor Abs were detectable in chimeras treated with Tregs at any time point, whereas control mice (BMT recipients receiving the same regimen without Tregs) developed substantial levels of antidonor Abs at late time points (data not shown). Thus, Treg-treatment prevents development of a humoral antidonor response in BMT recipients.

To further characterize the state of tolerance, *in vitro* MLR assays were performed. Hyporesponsiveness toward the donor was observed already 1 week post-BMT in Treg-treated recipients (Figure 4D) and was reduced to the level of self-reactivity during long-term follow-up (Figure 4E). Thus, Treg-treated chimeras demonstrate *in vivo* and *in vitro* tolerance.

### Intra-graft frequencies of mast cells and Tregs are increased in Treg-treated chimeras

**Figure 5:**
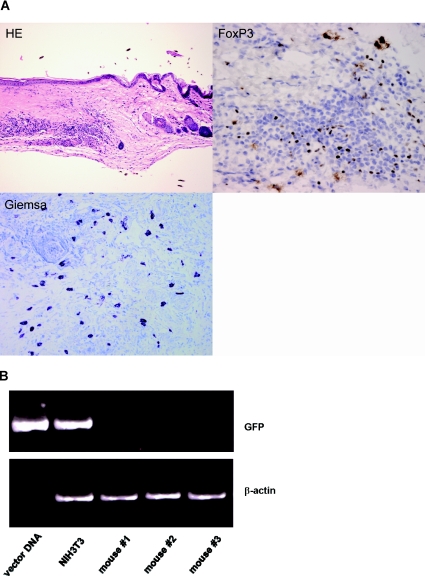
Donor skin grafts of Treg-treated chimeras show high frequencies of mast cells and Tregs. (A) Histopathology of donor skin grafts from Treg-treated chimeras revealed high frequencies of mast cells (Giemsa) and FoxP3 positive cells (immunohistochemistry) (HE, hematoxylin and eosin stain, magnification 100×; FoxP3, immunohistochemistry with specific FoxP3 antibody, magnification 400×; Giemsa, Giemsa staining, magnification 200×; representative graft shown 5 months post-BMT). (B) Genomic DNA isolated from skin grafts of FoxP3-Treg treated mice (n = 3) was subjected to PCR analysis specific for GFP. Grafts lacked detectable GFP expression, indicating that graft-infiltrating FoxP3 Tregs did not originate from the therapeutically administered Tregs. FoxP3-transduced NIH3T3 cells and FoxP3 vector were used as positive controls. DNA levels of GFP (upper panel) and β-actin (lower panel) are shown.

In the Treg groups, donor skin grafts remained macroscopically intact for the length of follow up. Histopathological analysis at the end of the observation period showed an intact epidermis without lymphocytic infiltrates, intact skin adnexa and weak-to-moderate lymphocytic infiltrates in the dermis. Donor grafts of Treg-treated chimeras were remarkable for an abundance of mast cells, which have been linked to Treg-mediated tolerance (35). The frequency of graft-infiltrating mast cells was significantly increased in Treg-treated mice over tolerant chimeras induced with a nonmyeloablative regimen in which maintenance of tolerance does not depend on regulation (36) (mean density of mast cells [5 HPF counted] 28.6, n = 7, vs. 17.9, n = 3 controls, p = 0.0467; Student's t-test) (Figure 5A).

As immunohistochemistry also revealed substantial numbers of FoxP3 positive cells within donor grafts (37) (Figure 5A), the question arose as to whether the graft-infiltrating FoxP3^+^ cells originate from the therapeutically administered Tregs. To address this question directly, we analyzed DNA isolated from donor skin grafts from FoxP3-Treg treated mice for GFP expression by PCR. No GFP expression was detectable in the skin grafts, indicating that the FoxP3 positive Tregs infiltrating the donor grafts did not descend from the Tregs therapeutically applied at the time of BMT (Figure 5B). Already 2 weeks after BMT (earliest time point analyzed), no GFP^+^ cells were detectable by flow cytometry in peripheral blood in FoxP3-Treg treated mice, hinting that FoxP3-Tregs did not persist/circulate in large quantities. PCR analysis at the end of follow up (>5 months post-BMT) showed absence of GFP^+^ cells also in lymphoid organs (bone marrow [BM], spleen, lymph nodes, thymus; not shown), indicating a limited life-span for transferred Tregs. Taken together, these data suggest that tolerance induced through Treg therapy with noncytoreductive BMT is associated with the presence of graft-infiltrating mast cells and of Tregs not originating from the transferred population.

### Chimeras induced through Treg treatment demonstrate partial central and peripheral deletion of donor-reactive T cells

**Figure 6:**
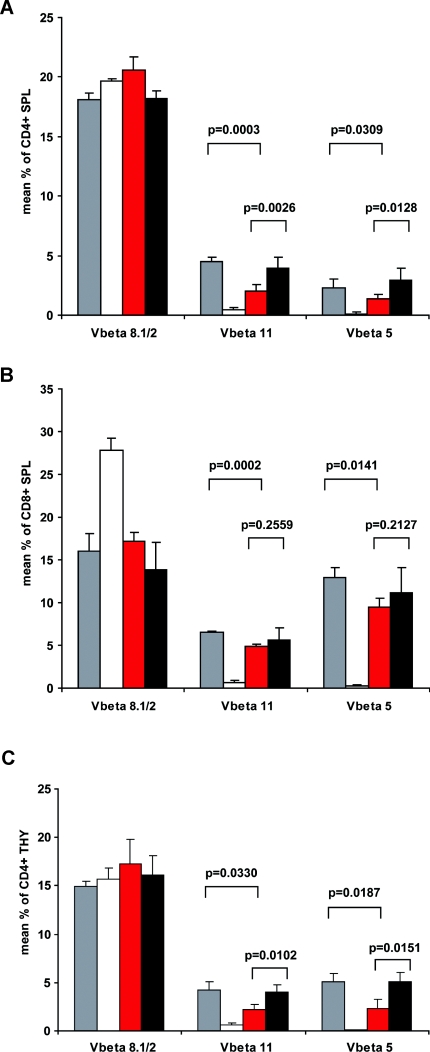
Chimeras induced through Treg treatment demonstrate partial central and peripheral deletion of donor-reactive T cells late after BMT. (A) Percentages of Vβ11^+^ and Vβ5^+^ (but not Vβ8^+^) CD4^+^ splenocytes (SPL) were significantly reduced in Treg-treated mice (red bars, n = 7) in comparison to naïve B6 mice (gray bars, n = 5; p = 0.0003 for Vβ11^+^, p = 0.0309 for Vβ5^+^) and in comparison to BMT recipients without Tregs (black bars, n = 6; p = 0.0026 for Vβ11^+^, p = 0.0128 for Vβ5^+^; 25 weeks after BMT). (B) CD8^+^ splenocytes (SPL) were also significantly deleted in Treg-treated chimeras in comparison to naïve B6 controls, indirectly indicating central deletion (as mature superantigen-reactive CD8 cells are not deleted extrathymically) (p = 0.0002 for Vβ11^+^, p = 0.0141 for Vβ5^+^). (C) Percentages of Vβ11^+^ and Vβ5^+^ (but not Vβ8^+^) single-positive CD4^+^ thymocytes (THY) were significantly reduced in Treg-treated chimeras (red bars, n = 3) in comparison to naïve B6 mice (gray bars, n = 3; p = 0.0330 for Vβ11^+^, p = 0.0187 for Vβ5^+^) and in comparison to BMT recipients without Tregs (black bars, n = 7; p = 0.0102 for Vβ11^+^, p = 0.0151 for Vβ5^+^; 22 weeks after BMT). Deletion was assessed by multicolor flow cytometry in selected mice. Gray bars denote naïve B6 controls, white bars denote naïve Balb/c controls. p values are shown for comparison between groups (two-sided Student's *t*-test), error bars indicate standard deviation.

Depending on the specifics of the chimerism model, both deletional (central and peripheral) and nondeletional mechanisms contribute to tolerance to varying degrees (7,36,38). In chimeras, the frequency of certain superantigen-reactive T-cell populations correlates with the deletion of ‘truly alloreactive’, donor-specific CD4 cells (as it can be assessed directly by employing TCR-transgenic T cells recognizing donor MHC [39]) and hence is a useful surrogate marker to measure whether deletion occurs (3,4). T cells expressing particular Vβ subunits (i.e. Vβ11^+^ and Vβ5^+^ in the strain combination used herein) recognize endogenous superantigen bound to the *donor* MHC class II allele I-E (40,41), which is not expressed in the recipient strain B6. No deletion was detected immediately after BMT in peripheral blood, spleen or thymus (∼2 weeks post-BMT in peripheral blood, 1 and 2 weeks post-BMT in spleen and thymus, not shown). However, we observed a significant deletion of CD4 cells in peripheral blood (data not shown) and spleen in Treg-treated BMT recipients late after BMT (25 weeks post-BMT) (Figure 6A). No such deletion was seen in control mice without Tregs. Deletion late after BMT was significant, but was not complete (compared to naïve Balb/c). CD8 splenocytes were also clonally deleted, providing evidence for intrathymic deletion taking place because mature superantigen-reactive CD8 cells—in contrast to CD4 cells—are not deleted extrathymically (as they do not efficiently bind to the superantigen-presenting MHC II), but only intrathymically at the double positive stage of development (Figure 6B). Central deletion was also directly evident among thymocytes of Treg-treated long-term chimeras (22 weeks post-BMT) (Figure 6C). The degree of deletion of CD4 splenocytes was more profound than that of CD8 splenocytes (54% vs. 27% deletion of Vβ11, p = 0.0028). This difference in the extent of deletion between CD4 and CD8—with CD4s being deleted significantly more extensively—suggests that peripheral deletion of CD4 cells also took place, in addition to central deletion occurring for both CD4 and CD8. Thus, intra- and extrathymic clonal deletion was evident in chimeras, but was not complete, revealing that both deletional and nondeletional mechanisms contribute to maintenance of tolerance in Treg-induced mixed chimeras.

## Discussion

The presented studies show that polyclonal recipient Tregs induce engraftment of conventional doses of allogeneic BM in recipients conditioned solely with costimulation blockers and rapamycin, leading to long-term mixed chimerism and donor-specific *in vivo* and *in vitro* tolerance. This unique protocol avoids all cytotoxic recipient treatment (irradiation, cytotoxic Abs and drugs).

The mixed chimerism approach resembles a two-edged sword. Its unparalleled effectiveness in achieving robust tolerance is counterbalanced by the toxicity of the drastic cytoreductive recipient preparation necessary for BM engraftment. Our results provide proof-of-concept that mixed chimerism and tolerance can be attained without toxic recipient preparation. Two of the remaining components of our regimen—rapamycin and CTLA4Ig (abatacept/belatacept) (42,43)—are already clinically available. Anti-CD40L, alas, is not and probably will not be any time soon. However, anti-CD40 mAbs have been used with success in chimerism and tolerance models (44,45) and they, or other alternatives, might eventually be substituted for anti-CD40L (46).

Treg therapy on its own seems most potent if hosts are lymphopenic (47) or if Tregs are designed to express a specific TCR (16,17,29). In contrast, in this study we used polyclonal Tregs from wild-type mice, and fully allogeneic recipients with an unperturbed T-cell repertoire. The remarkable effectiveness of Tregs in this model is conceivably facilitated by the short time span during which their action is required to induce BM engraftment. Once BM has engrafted, chimerism presumably contributes to maintenance of tolerance. Notably, all Treg populations tested—FoxP3-Tregs, nTregs and iTregs—achieved the same outcome, suggesting that this is a robust and reliable method for establishing mixed chimerism without cytoreduction. Pending further in-depth analysis into the three Treg subsets, it cannot be ruled out at present, however, that the different Treg populations achieve the same outcome through different mechanisms.

Up to now, it has been considered necessary to transplant clinically unobtainable high doses of allogeneic BM to achieve engraftment in recipients not receiving cytoreductive treatment (4,14,48). Our results suggest that engraftment of moderate doses of allogeneic BM is feasible if the immunological host-versus-graft barrier is sufficiently overcome. Treg therapy is uniquely potent in this respect, as previous attempts have failed to reach this goal through other interventions (49,50). Recently, chimerism was induced with the help of Tregs, however, 5 Gy total body irradiation was required (11). Such a sublethal dose of recipient irradiation is unacceptably toxic for organ transplant patients as it leads—among other side effects—to profound leukopenia and thus a substantial risk of life-threatening infections. Remarkably, a comparable magnitude of chimerism was achieved in our present studies with 2 × 10^7^ BM cells transplanted together with Tregs and rapamycin (plus costimulation blockade) as had previously been obtained with ten times as many BM cells (2 × 10^8^ BM cells) transplanted with costimulation blockade alone (4,14). Likewise, levels of T-cell chimerism—albeit low—were similar to what had been observed with 2 × 10^8^ BM cells without Tregs (4).

The transferred Tregs have a limited life-span (as indicated by results from experiments with FoxP3-Tregs co-expressing GFP) but nevertheless FoxP3^+^ regulatory cells infiltrate tolerated donor skin. Accumulation of mast cells in skin grafts also implies a role for regulatory mechanisms in the maintenance of tolerance. In Treg-treated chimeras, peripheral and central clonal deletion was evident but incomplete late after BMT, providing evidence that tolerance is maintained through a combination of deletional and regulatory mechanisms. Moreover, deletion progressed more slowly over time than in mixed chimeras of previous studies induced with nonmyeloablative conditioning (3). Early after BMT (∼2 weeks), when donor-hyporesponsiveness is evident in MLRs and donor skin is already accepted (unpublished data), no deletion of donor-reactive T cells is yet detectable in thymus and spleen. This is consistent with the hypothesis that the relative contribution of nondeletional mechanisms inversely correlates with chimerism levels (38) and previously published data showing that regulation has a more prominent role in the early induction phase after nonmyeloablative BMT (36). Taken together, our results suggest that transferred Tregs induce BM engraftment with unique potency in recipients with intact hematopoietic and immune systems. Chimerism having been established through Treg therapy, tolerance is subsequently maintained by both clonal deletion and nondeletional mechanisms apparently not requiring persistence of the transferred Tregs.

Translation of the powerful tolerogenic effects of mixed chimerism into widespread clinical use has so far been prevented by the risks inherent in the necessary cytotoxic recipient conditioning. Outlined in these studies is an approach that achieves mixed chimerism and tolerance without these risks.
